# The Examining Board and Its Critics

**Published:** 1896-12

**Authors:** 


### THE EXAMINING BOARD AND ITS CRITICS.

We call attention to the letter in this issue from Dr. Anthony, President of the Board of Examiners, in reply to a late criticism of the Board's performance of duty. Nothing could be clearer as to the definition of the Board's powers and duties than this statement by Dr. Anthony, unless it be the text of the law itself, which probably the self-appointed critic has never read. The Board has certainly accomplished a great deal already, and we do not doubt that it is doing all that it can within the law. Much more could be done, however, if those who volunteer their criticisms would exert their valuable energies and talents in assisting the Board and its attorneys in bringing offenders to justice.

---

ACCORDING to the Cincinnati *Lancet-Clinic*, the medical schools of the United States number about 175. Of these 120 are regular, 19 homeopathic, 7 eclectic, 2 physio-medical, and 12 unclassified. Eight are for women specially, five of these being regular, two homeopathic, and one eclectic. In eight of the other colleges women are permitted to matriculate, and four are exclusively for colored people.

